# Activity-associated miRNA are packaged in Map1b-enriched exosomes released from depolarized neurons

**DOI:** 10.1093/nar/gku594

**Published:** 2014-07-22

**Authors:** Belinda J. Goldie, Matthew D. Dun, Minjie Lin, Nathan D. Smith, Nicole M. Verrills, Christopher V. Dayas, Murray J. Cairns

**Affiliations:** 1 School of Biomedical Sciences and Pharmacy, Faculty of Health and Medicine, University of Newcastle, Callaghan, NSW 2308, Australia; 2 Schizophrenia Research Institute, Sydney, Australia; 3 Centre for Translational Neuroscience and Mental Health, Hunter Medical Research Institute, University of Newcastle, Callaghan, NSW 2308, Australia; 4 Hunter Cancer Research Alliance, Hunter Medical Research Institute, University of Newcastle, Callaghan, NSW, 2308, Australia; 5 School of Environmental and Life Sciences, University of Newcastle, Callaghan, NSW 2308, Australia; 6 ABRF, Research Services, University of Newcastle, Callaghan, NSW 2308, Australia

## Abstract

Rapid input-restricted change in gene expression is an important aspect of synaptic plasticity requiring complex mechanisms of post-transcriptional mRNA trafficking and regulation. Small non-coding miRNA are uniquely poised to support these functions by providing a nucleic-acid-based specificity component for universal-sequence-dependent RNA binding complexes. We investigated the subcellular distribution of these molecules in resting and potassium chloride depolarized human neuroblasts, and found both selective enrichment and depletion in neurites. Depolarization was associated with a neurite-restricted decrease in miRNA expression; a subset of these molecules was recovered from the depolarization medium in nuclease resistant extracellular exosomes. These vesicles were enriched with primate specific miRNA and the synaptic-plasticity-associated protein MAP1b. These findings further support a role for miRNA as neural plasticity regulators, as they are compartmentalized in neurons and undergo activity-associated redistribution or release into the extracellular matrix.

## INTRODUCTION

Post-synaptic excitation triggers a localized and temporally regulated cascade of protein synthesis, modification and other molecular activity, which culminates in the remodelling of dendritic spines’ size, shape and receptor density. These processes ultimately modify the strength of the neural connection, changing its potential for subsequent excitation from the same inputs, and are essential for encoding experience in the cellular networks of the brain. At the molecular level, this process is facilitated by the neurons’ capacity to organize localized, input-restricted protein synthesis within dendrites and dendritic spines (1). While mRNA coding these proteins are transcribed from DNA in the nucleus and distributed and stored locally throughout the soma until needed, little is known about the mechanisms directing dendritic mRNA transport and, more importantly, how translation is suspended until required (2). Evidence from the study of key neuronal genes such as CamKIIα (3), MAP2 (4), MBP (5) and β-actin (6) has demonstrated the role of localization elements (LEs) encoded in the 3′ UTR of the mRNA for binding proteins that “chaperone” the transcript through the cell. In each case, the RNA binding protein identified was unique to its target transcript; however with so much mRNA trafficking in neurons it seems unlikely that each one will have its own “personal” chaperone. It would be less cumbersome to have more redundant systems where multiple transcripts destined for the same location could be recognized by small “adaptors” to each transcript, which associate reversibly with their cargo and potentially respond to dendritic location and synaptic activation.

A strong candidate to provide this logistic support to mRNA trafficking is the class of 17–22 nucleotide short, non-coding transcripts known as microRNA (miRNA). These post-transcriptional regulators recognize their target mRNA by signatures in their 3′ UTRs known as miRNA Recognition Elements (MREs) that are only 6–8 nucleotides long; thus a single miRNA has the flexibility to regulate the expression of many mRNAs. In support of a neuron-specific trafficking role, many miRNAs are brain specific or brain enriched, and play critical roles in neuronal differentiation and morphogenesis (7,8). In experiments where miRNA biogenesis is impaired or ablated, the resulting phenotypes are grossly abnormal, exhibiting improper differentiation, incomplete neural patterning including reduced arealization and layering, lack of interneurons, and impaired connectivity, dendritic targeting and arborization (8–10).

miRNA utilize the Argonaute (Ago) family of RNA-binding proteins and provide the specificity component for their protein complex known as an RNA-induced silencing complex (RISC). Activated RISC molecules have been associated with a range of functions particularly gene silencing and RNA interference mediated by RNA destabilization (11). However, they are also thought to mediate interactions with the 5′ cap of mRNA, or even arrest ribosomes, to confer translational repression (12). The RISC has been demonstrated to play an important role in long-term potentiation (LTP) in *Drosophila*, as it decompiles in response to stimuli, releasing memory-associated transcripts that are subsequently actively translated (13); similarly in the rat, memory-associated transcripts were shown to be de-repressed at the synapse in an activity-dependent manner (14).

To further investigate the putative role of miRNA in regulating and segregating dendritic gene expression in mammalian systems, we examined the redistribution of miRNA and their mRNA targets in differentiated human neuroblasts in response to a stimulating concentration of K^+^ ions. These analyses revealed K^+^-associated changes in both miRNA and mRNA expressions; strikingly, modulation of miRNA was confined to the synapto-dentritic compartment, while mRNA followed no consistent pattern. Down regulation of miRNAs in the neurite fraction was accompanied by a corresponding release of MAP1b-containing microvesicles enriched for primate-specific mature miRNA. These data also suggest that small RNA species are subject to a functionally specific selection process for transmission or disposal in these vesicles.

## MATERIALS AND METHODS

### Cell culture

Populations of SH-SY5Y human neuroblastoma cells (ATCC, kindly provided by Jean-Marie Sontag, University of Newcastle) were maintained at 37°C, 5% CO_2_, 90% humidity in Dulbecco's Modified Eagle's Medium (DMEM, Hyclone) supplemented with 10% Fetal Calf Serum (FCS, Sigma Aldrich), 2% HEPES and 1% L-glutamine. Cells were routinely passaged and harvested by washing with phosphate buffered saline (PBS) followed by brief incubation with trypsin.

### Differentiation

To obtain neuronal cells, populations were seeded as noted in individual methods and differentiation was induced as follows. After 24 h (Day 0), medium was replaced with medium supplemented with 10 μM *all-trans* retinoic acid (ATRA, Sigma). Flasks were incubated wrapped in foil for 5 days; media was changed on Day 3. On Day 5, ATRA was removed by washing 3 times with DMEM before continuing with methods as described.

### Depolarization

Depolarization was induced by 3-min room temperature incubation in stimulating HBS (35 mM NaCl, 100 mM KCl, 0.6 mM MgSO_4_.7H_2_O, 2.5 mM CaCl_2_.2H_2_O, 10 mM HEPES, 6 mM Glucose) (15). After depolarization, HBS was replaced with warm complete medium and cells were allowed to recover for 10 min under culturing conditions.

Two depolarization regimens were employed using the above strategy. These included a single stimulus + recovery and four consecutive stimulus + recovery cycles, designed to mimic patterns of electrical activation required to induce early-phase and late-phase LTP respectively (16). In addition, sham-depolarized controls were prepared using a non-stimulating HBS from which KCl was omitted and NaCl was increased to 140 mM.

### Separation of neurites from cell bodies

Cells were seeded into high-yield flasks (HY flasks, Millipore) with a growth area of 600 cm^2^ to ensure enough RNA would be produced from the fractions. To obtain active neurites during differentiation, cells were harvested after four days of ATRA treatment, using a method modified from Meyerson (17). Depolarization conditions were prepared as described, harvested and washed twice with ice-cold EDTA buffer (0.54 mM EDTA, 137 mM NaCl, 10 mM Na_2_HPO_4_, 2.7 mM KCl, 0.15 mM KH_2_PO_4_ pH 7.4, 100 U/ml RNase inhibitor). Individual samples were resuspended in 10 ml ice-cold EDTA buffer and homogenized with 6 strokes at 60 rpm with a teflon/glass homogenizer (Potter-Elvehjem). The homogenate was then loaded onto a 3.5 ml cushion of 20% sucrose in EDTA buffer and centrifuged at 500 g for 4 min, 4°C. The “neurite” fraction was collected from the load/20% sucrose interface, and the pellet retained as the “cell body” fraction. Intact cells (1 ml) were also collected prior to fractionation as a whole-cell control. Fractions and whole-cell controls were purified by centrifugation at 15 500 g for 40 min, 4°C, and the pellets used for RNA extraction. Neurite fractions were characterized by quantitative real-time PCR (qPCR) enrichment of transcripts for synaptophysin (SYP) and GAP43 compared with cell body fractions, as described in Supplementary Materials (Supplementary Figure S1).

### Exosome purification

To achieve the large populations necessary to obtain enough vesicular RNA for multiple avenues of analysis, cells were seeded in high-yield flasks (HY flasks, Millipore) with a growth area of 1000 cm^2^. After differentiation, cells were depolarized once and the buffer retained for vesicle collection. Non-stimulating HBS from sham-depolarized controls was retained and processed alongside depolarization buffer to confirm that the release of vesicles was a direct result of depolarization and not residual from other sources such as fetal bovine serum. Cells were not allowed a recovery, and instead were immediately harvested and lysed in TRIzol for profiling of cellular miRNA remaining acutely after depolarization.

Exosome ultra-concentrate was obtained from the depolarization buffer by centrifugation through Amicon Ultra-15 100kD centrifugal filters (Millipore) as described (18). Briefly, buffer was centrifuged in 15 ml aliquots at 4000 rpm for 3 min at RT to a final volume of approximately 100 μl, which was passed through a 0.1 μm syringe filter to remove debris. Ultra-concentrate was then DNaseI treated (Invitrogen) and washed 3 × 15 ml (with centrifugation as before) with PBS before 1 ml of TRIzol was added for RNA extraction.

### Exosome collection from separated neurites and cell bodies

Neurite and cell body fractions were prepared as described above to the point of fraction collection. Separated fractions were then depolarized for 3 min with 100 mM KCl as described above before proceeding with the final 40 min centrifugation step. The supernatant was processed as described above for exosome purification. Exosomal RNA was extracted as described below, while exosomal protein was quantified using a bicinchoninic acid protein assay kit (Pierce) according to the manufacturer's instructions.

### Electron microscopy

Ultra-concentrated exosomes, prepared as described above, were fixed in 3% glutaraldehyde in 0.1 m phosphate buffer (pH 7.3) overnight at 4°C. The samples were air-dried on copper grids coated with formvar membrane made from 1% polyvynilformal 15/95 in ethylene dichloride, and stained with 0.5% uranyl acetate in 30% ethanol for 10 min and further stained with lead citrate for 10 min. Lead precipitates on grid sections were removed by rinsing in 0.05 M NaOH before further rinsing in distilled H_2_O. Grids were left at room temperature to dry and the stained sections were examined using a JEOL-1200EX transmission electron microscope (JEOL, Tokyo, Japan) operating at 80 kV.

### Western blotting

Western blotting was performed on exosomal protein extracted using modified RIPA buffer (1% SDS, 1% Triton X-100, 500 mM sodium fluoride, 50 mM EDTA, 10 mM sodium orthovanadate, 0.05% sodium deoxycholate) containing a protease inhibitor cocktail (Roche) at 100°C for 5 min, vortexed vigorously and centrifuged at 10 000 ***g*** for 15 min at 4°C. Quantification of the isolated protein supernatant was achieved using a bicinchoninic acid protein assay kit (Pierce) according to the manufacturer's instructions. 20 μg of exomsome protein was boiled in NuPAGE LDS Sample Buffer (4X) (Invitrogen) supplemented with 2% 2-mercaptoethanol for 5 min and resolved on 4–12% NuPAGE Bis-Tris Gels polyacrylamide gels (Invitrogen). The resolved proteins were then transferred onto nitrocellulose membranes under a constant current of 350 mA for 1 h. The nitrocellulose membranes were blocked overnight in 5% skim milk powder in TBS (Tris-buffered saline: 100 mM Tris/HCl, pH 7.6, and 150 mM NaCl) (pH 7.4) supplemented with 0.1% Tween 20 (TBST). Membranes were rinsed in TBST and probed overnight with primary antibodies against LAMP1 (1:1000) and FLOT1 (1:1000) (both Abcam) in 1% skim milk powder in TBST. Membranes were then further probed for 1 h with a 1:5000 dilution of horseradish peroxidase-conjugated secondary antibody at room temperature. Following a further 3 washes in TBST, cross-reactive proteins were visualized using an ECL (enhanced chemiluminescence) kit (GE Healthcare) according to the manufacturer's instructions.

### Mass spectrographic analyses

Cells and exosomes were lysed in RIPA buffer supplemented with protease and phosphatase inhibitors (as mentioned above) before resolution on 4–12% Bis-Tris acrylamide gels. The gels were then silver stained and bands excised. Peptides were generated using trypsin by in-gel digestion of excised bands of interest (19,20). Proteins were digested for 18 h at room temperature and peptides then analysed by tandem mass spectrometry (LC-MS/MS). Peptides sequenced using AmaZon ETD Ion Trap (Bruker Daltonik GmbH, Preston, VIC, Australia) with peptide separation achieved prior by PRLC using Dionex Ultimate 3000 RSLCnano (Dionex, Idstein, Germany). Files were converted into MASCOT Generic Format and imported into Bruker's ProteinScape platform (Bruker Daltonics, Bremen, Germany) for database searching. Searches were performed against the SwissProt database (version 57.15) using in house licensed MASCOT server (version 2.3.02, Matrix Science), with the species set to Homo sapiens and the number of allowed trypsin missed cleavages set to 2. Carbamidomethylation of Cysteine was set as a fixed modification, whereas oxidation of Methionine and phosphorylation of Serine, Threonine and Tyrosine were set as variable modifications. The parent ion tolerance was set to 1.4 Da with fragment ion set to 0.7 Da. Peptide thresholds were set requiring false positive rate less than 0.05% and a MASCOT score greater than 40. Those spectra meeting these criteria were validated by manual inspection to ensure accurate y- and b-ion detection with overlapping sequence coverage. MS was conducted from 3 separate experiments and peptides identified in each run are presented meeting the criteria above.

### RNA Extraction and QA

Total RNA was extracted using TRIzol (Invitrogen), with an enhanced overnight −30°C precipitation using glycogen (Sigma) as co-precipitant. RNA quality was checked via Bioanalyzer RNA 6000 Nano chip, while small RNA composition was determined by Bioanalyzer Small RNA chip (both Agilent).

### Expression Analysis

For miRNA expression, total RNA was labelled using a FlashTag Biotin HSR RNA labelling kit (Genisphere), hybridized to Genechip miRNA 2.0 microarrays (Affymetrix) and scanned with Affymetrix GeneChip Scanner 3000 7G. For exon/gene expression, total RNA was transcribed to cDNA and amplified using the Applause WT-Amp Plus ST kit, then fragmented and labelled with the Encore Biotin module (both NuGEN). Labelled cDNA was hybridized to GeneChip Exon ST microarrays and scanned as before. QC of microarray data was performed as per manufacturer's recommendations.

Data were analysed using Genespring GX software to determine differentially expressed mRNA and miRNA. Initial functional analysis was conducted on predicted targets of candidate miRNA using the functional annotation clustering (FAC) tool on the DAVID bioinformatics portal (21). FAC reports the significance of functionally related clusters via an Enrichment Score (ES), which is calculated as –log of the geometric mean of *P*-values of the terms in that cluster. Thus, an ES of 1.3 is equivalent to a mean *P*-value of 0.05, and *P* < 0.01 is indicated by ES > 2. We therefore considered clusters with ES>2 to be significant. Integrated functional analysis was then undertaken using Integrated Pathways Analysis (IPA) software. A target analysis was performed on the lists of miRNA altered in each condition; this was then paired with experimentally observed changes in mRNA expression in the same condition. Using fold-change data, inversely correlated miRNA–mRNA pairings were filtered and subjected to pathways and other functional analyses.

For vesicular samples, presence/absence of transcripts was determined by limits of experimental detection. From our experience, the limit of detection by qPCR for validation purposes corresponds to a raw signal intensity of approximately 30 on these arrays; however, we set the cut-off at 40 to allow a margin of error. Selected transcripts were confirmed by qPCR as outlined below; expression was defined as the level of detection above background (DABG) readings from no-template control wells.

### Quantitative real-time PCR (qPCR)

Forty cycles of real-time PCR was performed as previously described (22). Briefly, multiplex oligo(dT)-primed or specific miRNA-primed reverse transcription was carried out on 500 ng DNase-treated total RNA using Superscript II reverse transcriptase (both Invitrogen) in a final volume of 20 μl. Real-time PCR was performed in triplicate 12.5 μl reactions on diluted cDNA with Power SYBRGreen master mix using an ABI Prism 7500 sequence detection system (both Applied Biosystems). For vesicle samples, 50 ng was reverse transcribed in half-volume reactions and treated as 1:5 dilution of cDNA in the PCR, which was carried out as above.

### Normalization

Gene expression qPCR was normalized to GUSB. The small RNAs used to normalize miRNA expression studies, however, are preferentially enriched in the nucleus, precluding conventional normalization. We therefore assessed data reproducibility by calculating the Co-efficient of Variation (CV) for each of the reference small RNAs (U6, U44, U49) and test miRNA in both cell body and neurite fractions. The CV is calculated as σ/${\bar{x}}$ and expressed as a percentage, and is a normalized measure of the spread of data. Variability ranged between 0.016 and 0.183 cycles; as a single PCR cycle relates to a 2-fold change in expression, this translated to 1.005-fold to 1.065-fold variability in expression. We then compared the variability within the reference small RNAs to the test miRNA and found no significant difference either in the cell bodies (*P* = 0.72) or neurites (*P* = 0.47). Finally, we confirmed the tightness of the data by checking for outliers using Grubb's test. No outliers were detected in either fraction at a significance cut-off of 0.01.

## RESULTS

### miRNA are down-regulated in response to depolarization in whole cells

**Figure 1.**
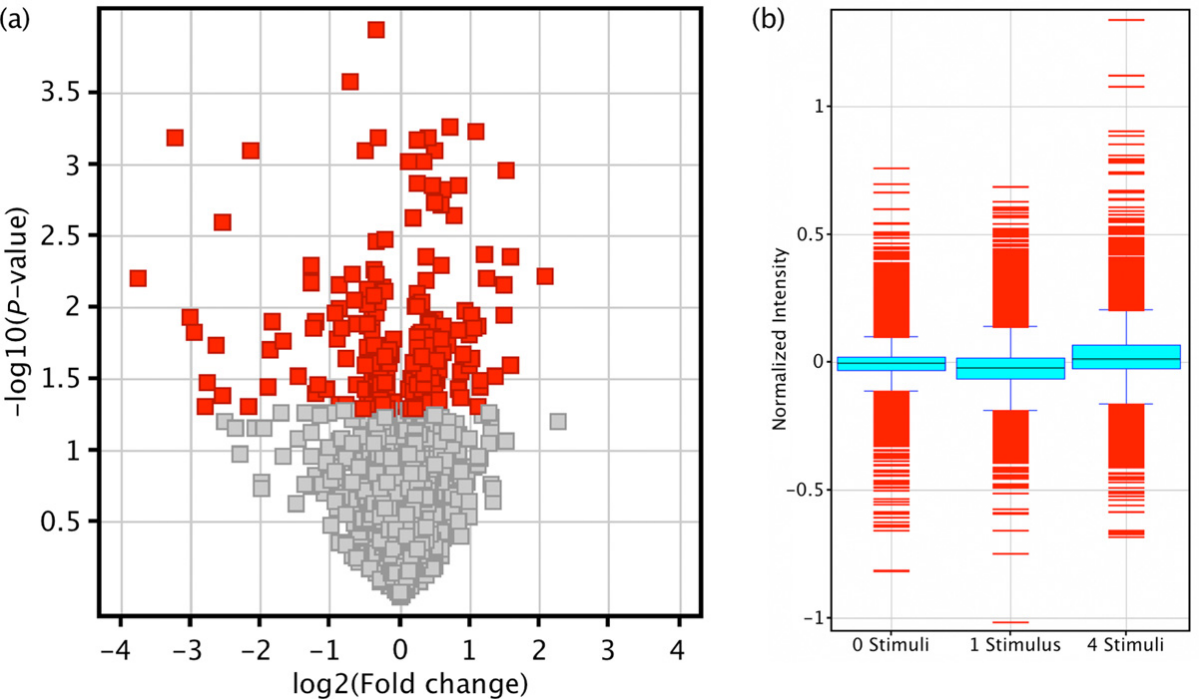
miRNA and mRNA responses to depolarization in whole cells. miRNA and mRNA microarrays were performed on 3 biological replicates of resting cells and cells subjected to one single or four sequential K^+^ depolarizations. (**a**) Volcano plot comparing miRNA expression between resting and single-depolarized cells demonstrates the bias for down regulation of miRNA in response to activity. Each square represents a single miRNA plotted by fold change (*x*-axis) and significance (*y*-axis). (**b**) Boxplot of mRNA microarray data. Compared with resting cells (left), the mRNA profile of cells was reduced by a single stimulus (centre). After 4 successive depolarizations (∼1 h), mRNA levels had recovered, and in fact exceeded that of resting cells (right).

Differentiated human SH-SY5Y neuroblast cultures were depolarized with stimulating levels of K^+^, either once or four times sequentially prior to fractionation and genome-wide miRNA and mRNA analysis. In whole cells, a single stimulus significantly altered the expression of 154 mature miRNA (*P* < 0.05), with a strong trend towards down regulation (71%) (Figure 1a). After 4 stimuli this was reduced to 99 altered miRNA, with 60% down-regulated. Between the 2 modes of depolarization, only 15 altered miRNA were common, and these also showed a bias for down regulation (66% down).

### Neurite-associated miRNA are down-regulated after depolarization

**Figure 2.**
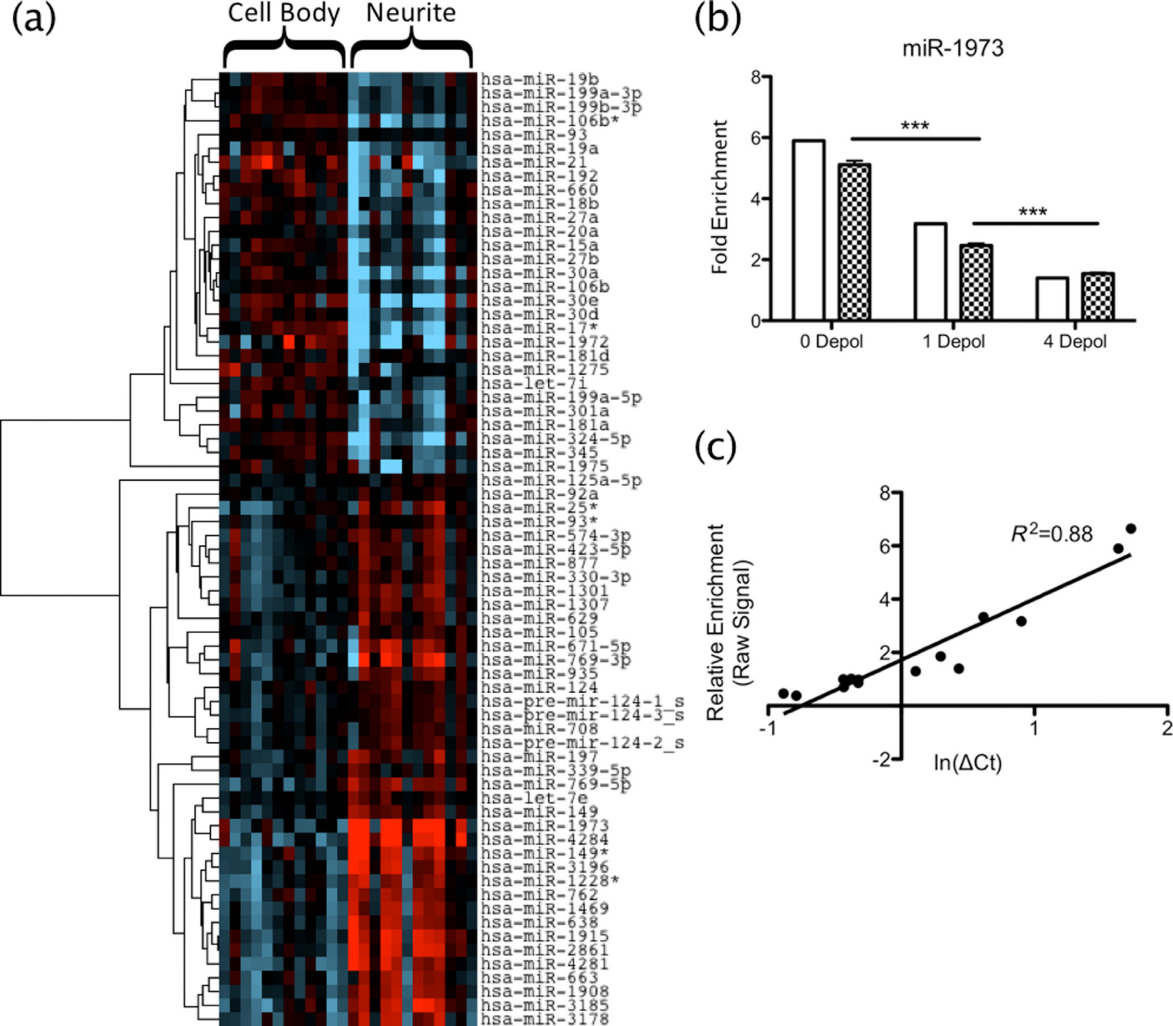
miRNA compartmentalization in neurites. (**a**) T-test was used to find miRNA differentially expressed between compartments (*P* < 0.05), independent of depolarization. Heat map shows supervised clustering of miRNA expression in neurites versus cell bodies. Two main clusters are revealed, indicating miRNA with low (blue) and high (red) expressions in neurites compared with cell bodies. (**b**) qRT-PCR results (chequered bars) closely matched those seen by microarray (open bars), both in enrichment and response to depolarization; miR-1973 shown is representative of 5 miRNA validated across 3 depolarization statuses (15 samples total). (**c**) Correlation of microarray and qPCR expression had *R*^2^ = 0.88, validating the microarrays.

**Figure 3.**
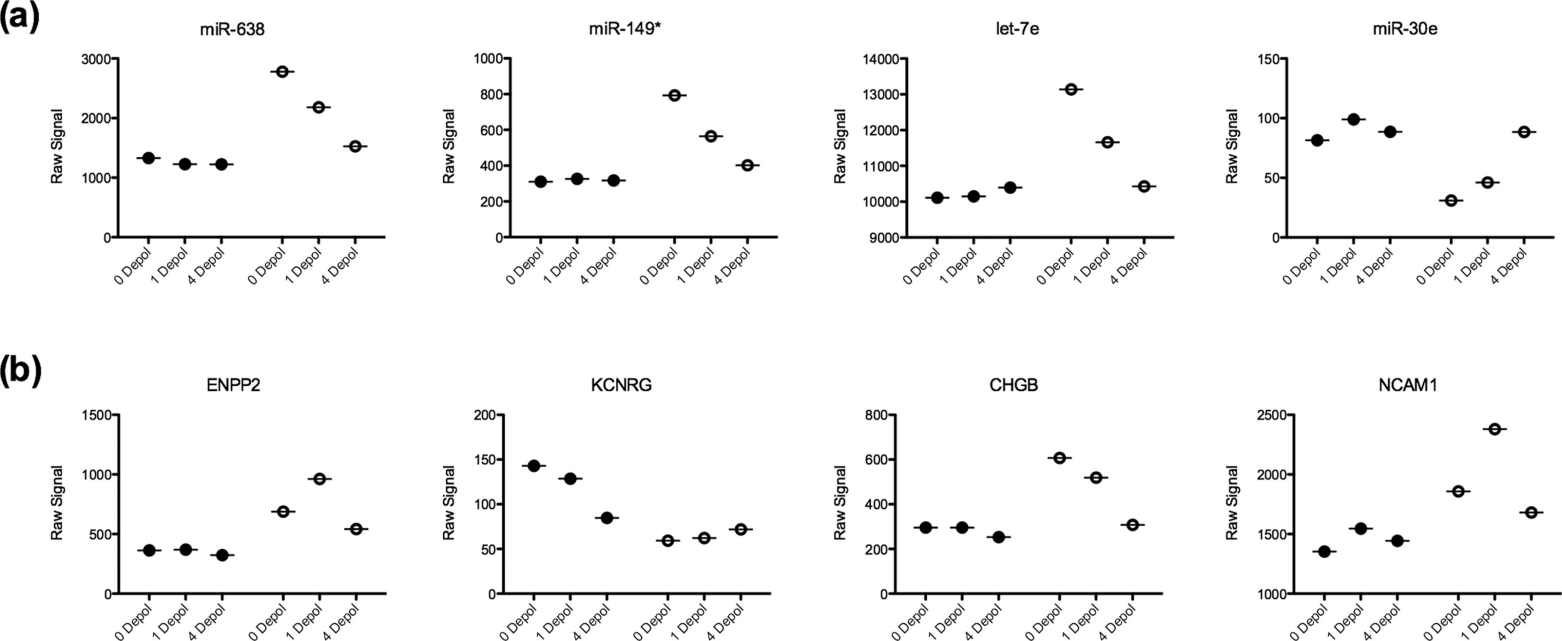
Different subcellular miRNA and mRNA responses to depolarization. Microarray data were analysed by two-way ANOVA on compartment and depolarization pattern to investigate the subcellular response to depolarization. (**a**) miRNA showed little response to depolarization in the cell bodies, however there were clear up- and down- regulation patterns in the neurites. (**b**) Comparatively, mRNA expression followed no consistent pattern in either fraction with successive depolarizations. Closed circles represent cell body samples; open circles represent neurites. 0 depol, 1 depol and 4 depol indicate resting, single depolarized and 4 sequential depolarized cells, respectively.

To further investigate the subcellular distribution of this depolarization-associated change in miRNA expression, we also analysed the somato-dendritic fraction and dendrite depleted cell bodies from resting and depolarized cells. These analyses reveal that miRNA appear to be compartmentalized in neurites with significant enrichment and depletion of 29 (*P* < 0.05) and 40 molecules (*P* < 0.05) respectively, including all 3 miR-124 precursor hairpins (Figure 2a). qPCR closely validated the array expression of miRNA tested (Figure 2b, miR-1973 representative) with *R*^2^ = 0.88 (Figure 2c). Interestingly, the neurite compartment responded to depolarization by up- or down-regulating miRNA expression (Figure 3a), in contrast to the cell body fraction, which did not display a significant response to stimulation with K+ ions (Figure 3b).

### miRNA are released from the neurite fraction in exosomes during depolarization

**Figure 4.**
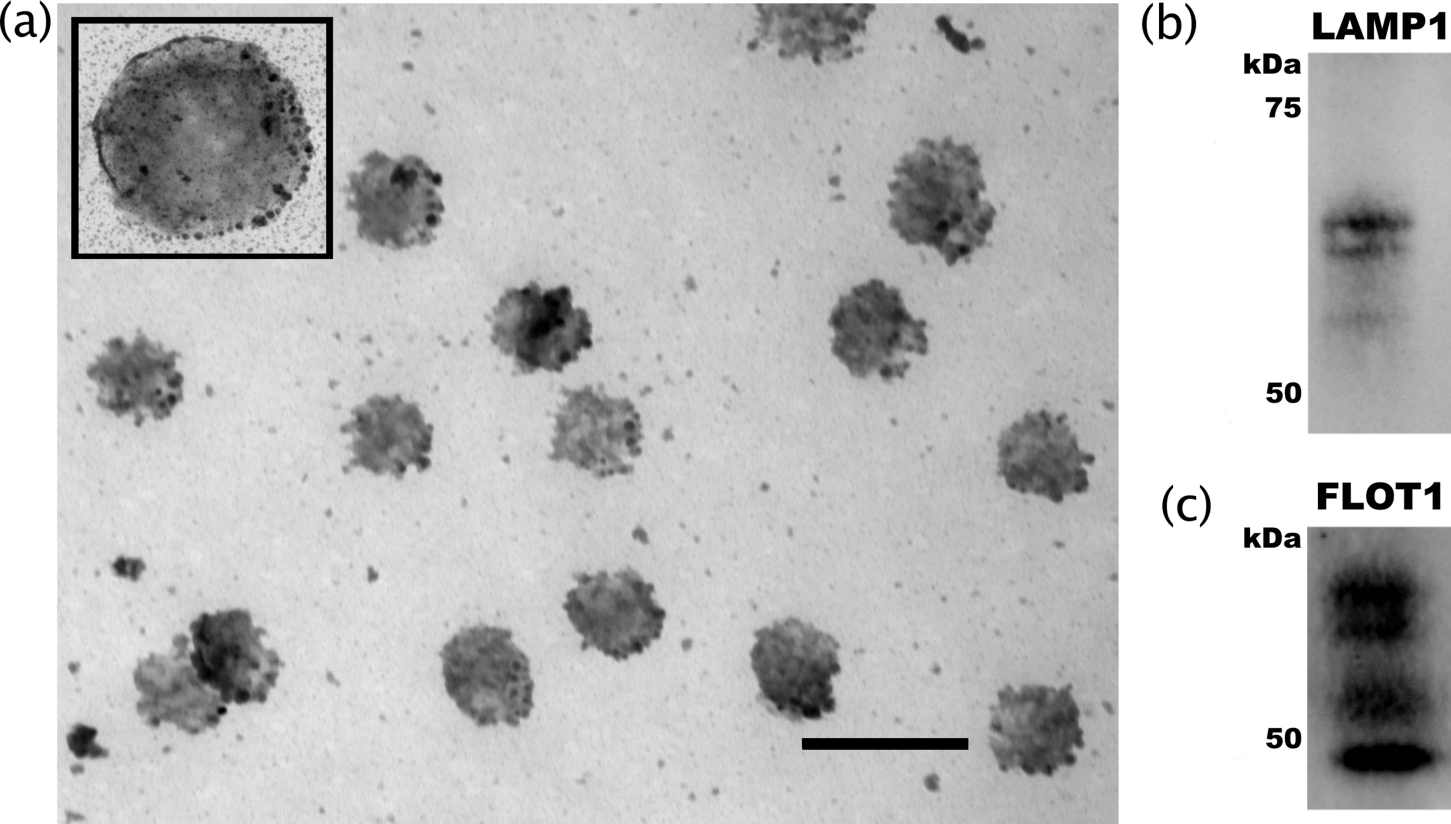
Structural characterization of vesicles as exosomes. Characterization of vesicles released from neurites following potassium-induced depolarization. (**a**) Glutaraldehyde-fixed samples were sliced and photographed using a JEOL-1200EX transmission electron microscope. Scale bar = 200 nm for main photo, 100 nm for inset. Analysis of particle size using ImageJ software found the average diameter of these vesicles to be 106 ± 7.62 nm. (**b**) Western blot analysis of exosome markers LAMP1 and (**c**) Flot1. Each experiment was replicated 3 times and representative blots are depicted.

**Figure 5.**
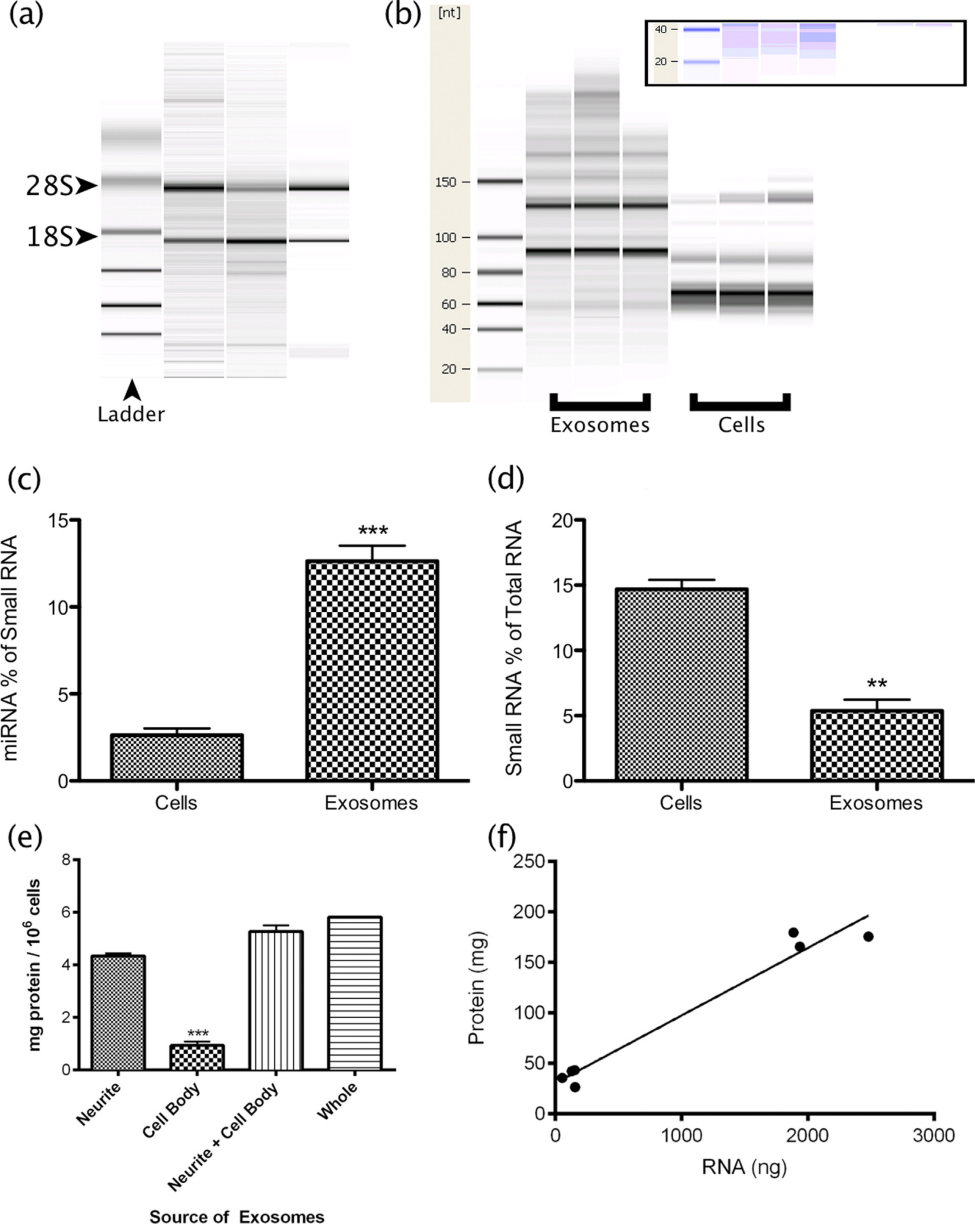
Vesicular nucleic acid composition is consistent with exosomes. (**a**) Bioanalyzer analysis of triplicate samples revealed the presence ribosomal RNA. (**b**) Bioanalyzer comparison of small RNAs derived from vesicles and cells. The vesicular samples appeared enriched for small RNAs (inset). (a and b) Lanes represent individual samples. (**c**) Quantitative analysis of Bioanalyzer data confirmed that miRNA comprised a significantly greater proportion of small RNA in the vesicles, despite (**d**) a reduced overall contribution of small RNA to total RNA. (**e**) Neurites and cell bodies were separated prior to depolarization. Quantitation of protein from vesicle fractions obtained indicated that the neurites are the primary source of depolarization-associated vesicle release. (**f**) Correlation of protein and RNA yields from neurite- and cell body-derived vesicles (*R*^2^ = 0.96).

The apparent depletion of somato-dentritic miRNA following depolarization did not lead to redistribution deeper in the soma as the abundance of individual miRNAs tested did not increase in this fraction (Figure 3). We thus speculated that these miRNA are degraded or disposed of in some other manner. One possibility is that miRNA are released from the dendrites as encapsulated microvesicles or exosomes in response to excitation (23). Analysis of the depolarization media supported this hypothesis, with EM revealing the presence of distinct vesicles (Figure 4a) of average diameter (106 ± 7.62 nm), which is at the upper end of the size range reported for exosomes (24,25). However, since the vesicles passed through a 0.1 μm filter their actual size is likely smaller and thus within range. The vesicles were positive for exosome markers LAMP1 and Flotillin1 (Figure 4b and c), and contained ribosomal RNA (Figure 5a) and a small RNA population significantly enriched for miRNA (Figure 5b–d).

Importantly, 50% of the miRNA detected in the exosomes were enriched in neurites compared with cell bodies (Table 1). In particular, 4 of these miRNA (miR-638, -149*, -4281 and let-7e) were negatively regulated by repeated depolarization in this compartment. The vesicular miRNA cohort was also enriched (10/24) with primate-specific molecules (Table 1), which suggested that these depolarization-associated vesicles might have functional significance in the primate brain.

**Table 1.** miRNA detected in exosome samples

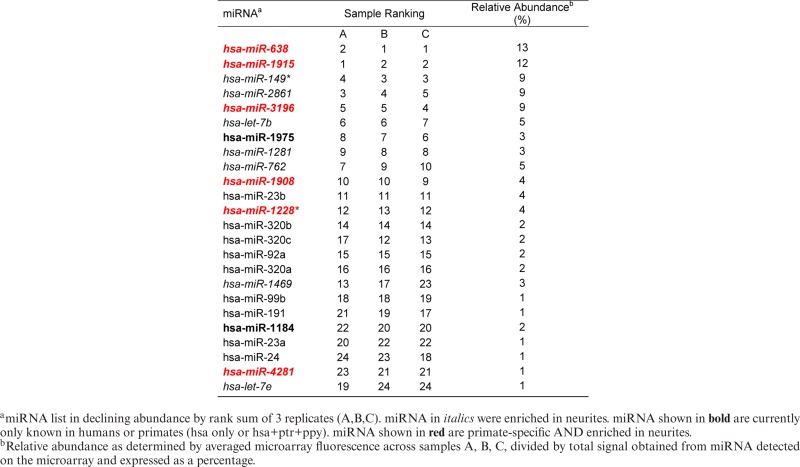				

Investigation of localization of vesicular release by separate depolarization of the neurites and cell bodies implicated the neurites as the primary source of RNA-containing vesicles. On average, 4.68-fold more vesicular protein was obtained from neurites (Figure 5e). RNA yield correlated significantly with protein (*R*^2^ = 0.96, *P* < 0.0001, Figure 5f), however neurite-derived samples contained 3.1 times as much RNA/mg protein, suggesting the possibility of a fraction-specific packaging mechanism. Supporting this, LAMP1 mRNA was significantly enriched in the neurite fraction and depleted from this fraction by depolarization (Supplementary Figure S2), suggesting site-specific synthesis of the exosomes.

### Exosomes are enriched with synapto-dendritic proteins depleted from depolarized cells

**Figure 6.**
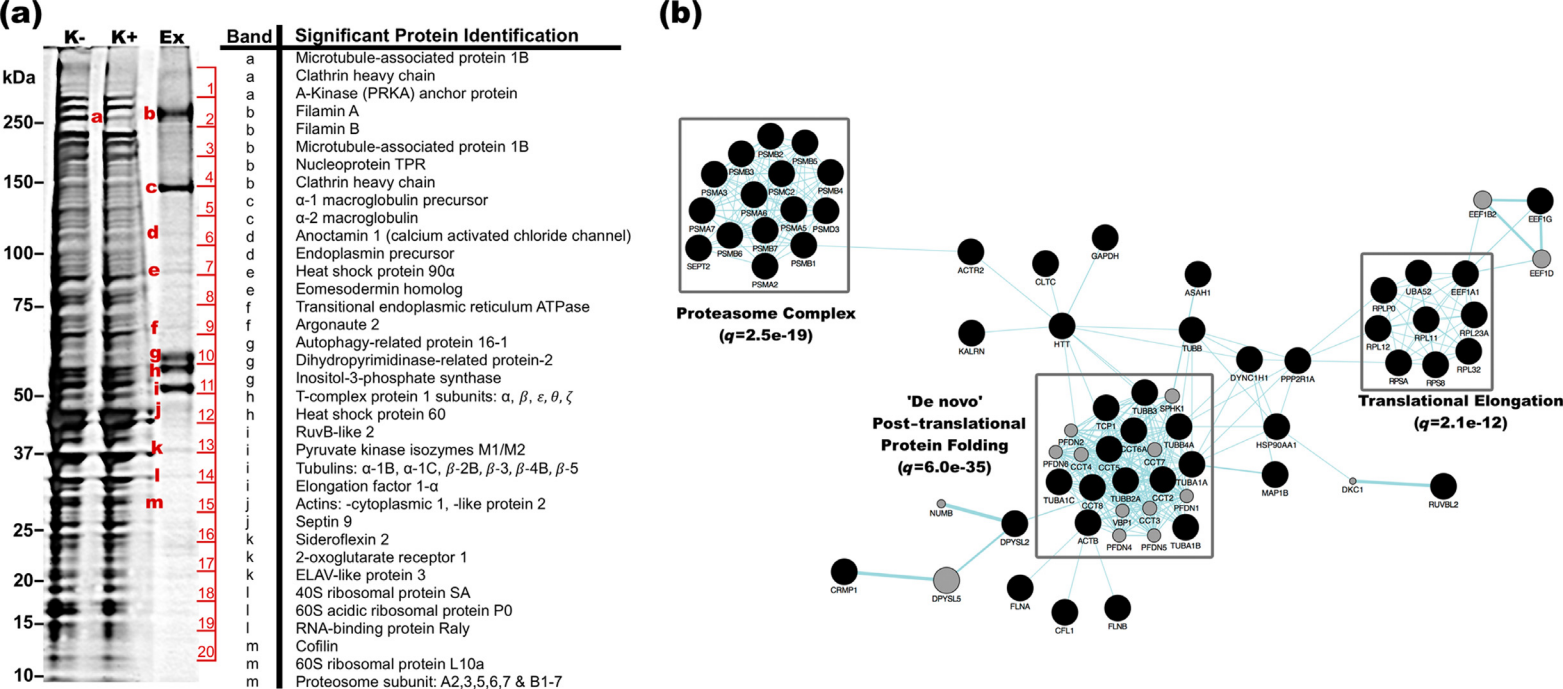
Proteomic analysis of exosomes. (**a**) Protein from resting cells (K^−^), depolarized cells (K^+^) and exosomes was solubilized and resolved by SDS-PAGE. Bands of interest (enumerated) were excised and sequenced by mass spectrometry. (**b**) Pathways analysis of 387 proteins identified by mass spectrometry. Analysis was performed using the Genemania plugin for Cytoscape software. Clusters representing the most significantly enriched functions are indicated by grey boxes, and indicative *q*-values shown.

Total protein from resting cells, depolarized cells and exosomes was visualized by silver-stained SDS-PAGE to investigate relative composition. The constitution of exosomes was unique compared with cells, while depolarized cells showed strong homology with resting cells. Strikingly, a band of approximately 250 kDa, present in resting cells, was strongly depleted by depolarization and appeared enriched in exosomes (Figure 6a, bands a and b). These bands were excised and analysed by mass spectrometry, identifying 15 proteins in the depolarized cell sample and 13 in the exosomes (Supplementary Table S1). Among these, 8 proteins were common, the most significant of which was microtubule-associated protein 1b (MAP1B, MASCOT score 4962). MAP1b is associated with synaptic plasticity, highly localized in the axon growth cone and dendritic spines, and displays activity-dependent pattern of translation in neurons (26–29). Interesting among proteins unique to the exosomal band were the two filamins, A and B (MASCOT scores 421.7 and 152.6 respectively). Filamins remodel the actin cytoskeleton, and filamin A is localized to the dendritic shaft (30), while filamin B has been associated with PSD95 (31).

### Proteomic characterization of exosomes by mass spectrometry

Twenty bands corresponding to the most abundant exosomal proteins (enumerated Figure 6a) were subjected to liquid chromatography mass spectrometry, positively identifying 1329 peptides representing 387 redundant proteins, approximately one third of which have been previously associated with exosomes (Supplementary Data S1); including the exosomal marker LAMP1 (Figure 4b). Gene ontology analysis (Supplementary Data S2) revealed markedly significant enrichment of the ‘De novo’ post-translational protein synthesis (*q* = 6.0e-35), proteasome complex (*q* = 2.5e-19) and translational elongation (*q* = 2.1e-12) pathways (Figure 6b). In particular, a strong bias was observed towards HSP90-CCT chaperone complex constituent proteins as well as known peptide clients of this structure (Supplementary Data S1, highlighted). Exosomal constituents were also significantly associated with a variety of neurological disorders including Parkinson's (*p* = 1.93e-08), Alzheimer's (*p* = 7.43e-04) and dementia (*p* = 5.19e-04), and IPA functional analysis generated a highly connected neuronal network among these proteins (Supplementary Figure S3).

### Convergent influence among depolarization-associated miRNA from whole cells

To determine whether the change in miRNA availability would have functional relevance in depolarized neuroblasts, functional analysis of predicted targets for all 5 common (single and multiple) up-regulated miRNA was performed. The intersection of these 5 predictions contained 24 genes, which were then subjected to FAC. Similarly, this analysis was also carried out for the 10 common down-regulated miRNA (192 target genes). This very stringent analysis of putative convergent miRNA influence among the up-regulated miRNA revealed tissue specificity for the brain (13/24 genes, *P* = 0.036), which also ranked top among targets for the 10 common down-regulated miRNA (94/192 genes, *P* = 0.006). The small gene list sizes restricted the number of significant clusters, however functionally relevant terms such as “post-transcriptional regulation of gene expression” (*P* = 0.003) and “central nervous system axonogenesis” (*P* = 0.008) were significant among targets of the down-regulated miRNA. These included key neuronal genes such as glutamate (GRIN2A) and GABA (GABRA1) receptors, neurexin3 (NRXN3) and doublecortin (DCX), as well as the schizophrenia-associated gene DISC1. Interestingly, the miRNA biogenesis gene DICER was the most strongly regulated predicted target of the down-regulated miRNA.

### Acute activation alters regulation of GPCR signalling pathway; chronic activation modulates regulation of transcription and translation

A single K^+^-induced depolarization also resulted in an acute reduction in whole-cell mRNA levels, with 251 changed genes after 1 depolarization, 74% of which were significantly down-regulated (*P* < 0.05, FDR). After 4 stimuli, cellular mRNA levels recovered and exceeded resting levels with 89% of 1168 differentially expressed genes being up-regulated (Figure 1b). FAC analysis of genes altered by 1 depolarization found only 1 functional cluster with ES>2, which was represented by terms involving GPCR signalling (ES = 3.19). After 4 depolarizations, 6 clusters had ES>2, and the focus shifted to the nucleus (ES = 7.3), RNA processing and splicing (ES = 4.4), ribosome biogenesis (ES = 3.1) and regulation of translation (ES = 3.1).

IPA software was used to integrate miRNA and mRNA expression to determine functional clusters of miRNA–mRNA regulatory relationships that are altered by depolarization. In response to single stimulus, this analysis confirmed the FAC results, demonstrating significant alteration of a regulatory network involving G-protein coupled receptor signalling (*p* = 1.36e-03, Supplementary Figure S4a), importantly indicating modulation of the cascade from receptor to nucleus. This finding was elaborated by revealing the significance of calcium-induced cAMP signalling via Gs-coupled receptors (*p* = 5.65e-06, Supplementary Figure S4b). Analysis of the response to repeated stimuli also agreed with the FAC analysis. A highly connected “DNA transcription” regulatory network (Supplementary Figure S5), centred on miRs-548 and -506, was identified among negatively correlated genes and miRNA (genes up, miRNA down).

### miRNA appear functionally compartmentalized in neurites

Predicted targets of miRNA that appeared compartmentalized within or excluded from neurites were submitted to DAVID for FAC as before. Targets of neurite-enriched miRNA comprised 5 functional clusters with ES>2, and suggested a compartmental requirement for increased regulation of transcripts whose products are involved in GTP and PKC signalling (ES = 3.7 and 3.0 respectively), axon guidance and development (ES = 2.5) and secretion of vesicles (ES = 2.4 and 2.3). Neurite-depleted miRNA also demonstrated functional specificity to this compartment with 3 functional clusters having ES>2. Predicted targets were enriched for regulation of pre- and post-translational modulation, specifically RNA-mediated gene silencing (ES = 2.4), and ATP-driven phosphorylation processes (ES = 2.4), including MAPK- and CAMK- family members and BDNF receptor NTRK2.

### Functional annotation of dendritic miRNA–mRNA interactions in response to depolarization

We next examined the subcellular response to activity by 2-way ANOVA. Depolarization significantly altered both the miRNA and mRNA composition of the neurites (*P* < 0.05, FDR), but strikingly the modulation of miRNA expression appeared localized to the neurites. Over successive depolarizations, the abundance of significantly changed miRNA transcripts remained almost unchanged in the cell body fractions while either increasing or decreasing in the neurites (Figure 3a). However, this was not the case for mRNA expression, which varied in both compartments (Figure 3b).

Integrated analysis with IPA found functionally relevant regulatory networks after 1 and 4 depolarizations. Most interesting among these was a “neuronal signalling” network, comprising 4 miRNA and many neurotransmitter receptors with negatively correlated response (mRNA up, miRNA down) after 1 stimulus (Supplementary Figure S6).

### Exosomes released during depolarization are enriched with primate specific miRNA

Since exosomal vesicles have been shown to contain various RNA species, we extracted and analysed total RNA to characterize this population. Strong ribosomal RNA bands were detected (Figure 5a), while the small RNA composition of these vesicles proved quite different from that of whole cells (Figure 5b), with stronger bands apparent at the sizes of transfer RNA and/or precursor miRNA transcripts. Moreover, miRNA comprised a significantly higher proportion of small RNA isolated from vesicles (Figure 5c, and visible in Figure 5b enhanced gel inset), despite a significantly smaller proportion of small RNA being present (Figure 5d).

Vesicular RNA from three separate cell populations was then analysed by microarray to investigate the profile of miRNA packaged in these particles. We detected the same 23 mature miRNA in all replicates, 9 (39%) of which were specific to humans or primates (hsa only, or hsa+ptr+ppy), and 14 (61%) were preferentially enriched in the neurites of their parent cells (Table 1). Moreover, 6 miRNA (26% of the vesicular cohort) fulfilled both criteria, suggesting that these dendrite-derived activity-associated exosomal miRNA have emerged recently in evolutionary history (Table 1).

For further confirmation of the microarray analysis, we examined the expression of 18S rRNA and miR-638, the most abundant miRNA detected in the exosomes by qPCR. This analysis included two additional samples that were not profiled by microarray. The transcripts were detected significantly above background (*P* < 0.0001), with 18S rRNA and miR-638 emerging at cycles 26.2 and 10.5 respectively, confirming the presence of both transcripts in the vesicles analysed by microarray as well as the novel samples.

### miRNA responding negatively to depolarization in neurites were released in exosomes, and target functionally relevant mRNA

More than 60% of the miRNA detected in exosomes were enriched in neurites compared with cell bodies, suggesting that they were derived from the dendritic fraction of the parent cells. In particular, 4 of these miRNA (miR-638, -149*, -4281 and let-7e) were negatively regulated by repeated depolarization in this compartment. We therefore wondered what the functional significance of encapsulating and releasing these 4 miRNA would have for the parent cells or surrounding cells that are the likely recipients of this molecular cargo? A list of mRNA potentially regulated by these miRNA collectively was compiled. This list comprising 95 transcripts, a significant proportion of which demonstrated tissue specificity for the brain (57/95, corr. *P* = 0.022), was submitted for FAC. Enrichment scores were slightly lower due to the small list used for analysis, however the terms significantly enriched (ES = 1.92–1.14) all related to synaptic function including synapse, post-synaptic density and regulation of neuronal synaptic plasticity.

## DISCUSSION

The findings presented here demonstrate that K^+^-induced depolarization significantly alters the miRNA composition of cultured human neurons at both the cellular and subcellular levels. The over representation of down-regulated miRNA expression suggests that neural activity leads to global reduction in post-synaptic gene silencing which could facilitate a rapid increase in local translation. This finding is supported by the work of Konopka and colleagues, which demonstrated enhancements of learning and memory in mice via miRNA depletion (32). Although fewer in number, a proportion of miRNA was also increased in depolarized cells and the somato-dendritic fraction suggesting that there is also some selective increase in gene silencing. Indeed, specific miRNA have been shown to be down- and up-regulated in neuronal plasticity in response to different stimuli (33). The predicted targets of both the down- and up- regulated miRNA were functionally clustered, revealing enrichment of genes involved in plasticity-related processes including synaptic activity, protein localization and neuron morphogenesis. The functionality of predicted target genes was supported by the observed genome-wide changes in gene expression.

These observations raise important questions about miRNA partitioning within the differentiated neuron and how it is so rapidly altered within minutes of depolarization. In respect to compartmentalization, the literature suggests that mature miRNA are transported to dendritic spines and the post-synaptic milieu within ribonucleoprotein complexes or granules—perhaps associated with their synaptically localizing mRNA targets (34,35). They may also become localized to the synapse in their inactive precursor form and become activated by maturation through dendritically localized Dicer protein that has been shown to itself be activated after excitation-dependent proteolysis by the calcium-dependent protease Calpain (36). More recently, the same group have shown that there is also post-synaptic localization of microprocessor complex proteins Drosha and DGCR8 capable of localized processing of primary miRNA transcripts (37). While it is unlikely that the changes we observed are due to changes in ribonucleoprotein trafficking of miRNA, an elevation of functional Dicer in the post-synaptic compartment could lead to the production of more mature miRNA in a matter of minutes after depolarization. As most of the miRNA were decreased after depolarization, it is plausible that these molecules were degraded by nuclease, perhaps as a secondary consequence of RISC decomposition brought about to facilitate activity-associated induction of translation (13,14).

An alternative explanation is that these molecules are packaged and released from the depolarized cells in vesicles. To test this hypothesis, we collected and analysed exosomal miRNA and identified a number of molecules that were enriched in the synaptodendritic fraction and depleted from this fraction in the parent cells after depolarization. The vesicular RNA was found to be enriched with small RNA, and surprisingly rich with recently evolved primate-specific miRNA. Depolarization of SH-SY5Y has previously been shown to trigger formation and release of synaptic vesicles (LDCVs, 200nm diameter), as well as a population of smaller, uncharacterized vesicles (60 nm diameter) (38–40). Combined with the present findings, these smaller vesicles are most likely exosomes, which have been found to be released from cultured cortical rat neurons (23), and are increasingly associated with intercellular miRNA trafficking, the establishment of polarity (reviewed in (24,41)) and extracellular distribution of mRNA and proteins (42), and have been shown to effect changes in target cell phenotype and activation (43).

These vesicles were only released from cells upon stimulation, suggesting an activity-specific function. This interpretation was supported by the target gene pathway analysis of the vesicular miRNA that was enriched with synaptic processes including synapse, post-synaptic density and regulation of neuronal synaptic plasticity. While it has been known for some time that recycling endosomes contribute to spine growth in response to LTP-inducing stimuli (44), the mechanisms of synaptic exosome release have begun to be elaborated in more detail in the *Drosophila* nervous system (45). Indeed, it is broadly suggested that exosomal transfer of miRNA may contribute to the function of neural systems by modifying local neurons and supporting cells such as astrocytes, oligodendrocytes and microglia. In support, Morel et al. (2013) have recently shown that miR 124a is released from neuronal exosomes and can regulate the expression of astroglial glutamate transporter (GLT1) (46). Moreover, exosomes originating from the pre-synaptic terminal were found to regulate retrograde signalling in their post-synaptic partners at the *Drosophila* neuromuscular junction via transfer of synaptotagmin4 (47). Their potential significance in synaptic function is supported by the MS-observed enrichment of MAP1B in these vesicles. MAP1B positive post-synaptic terminals were reported by Kawakami et al. to contain clear vesicles (26) and by Kitamura and colleagues as being associated with increased synaptogenesis (27). If these exosomes were transferred to neighbouring synapses during depolarization, the infusion of MAP1B and other substrates for spine remodelling, such as actin, tubulin and cofilin, would foster strengthening of the post-synaptic membrane. This is reinforced by the presence of Filamins A and B in exosomes. Although the Filamins have been shown to decrease in expression during development, the decrease is modest and they are still well expressed in the mature brain, in particular in the cortex (30,48). Filamin A has been shown to be localized to the dendritic shaft, but absent from dendritic spines, which has been suggested to enable the flexibility required for plasticity, and is also associated with surface localization of certain receptors, in particular several subtypes of potassium channel (30), which is interesting in the current context of potassium depolarization.

Conversely, it is possible that activity-associated exosomal release provides a means for cells to rapidly eliminate regulatory and structural molecules that are limiting or repressing the expansion of their post-synaptic function. This concept accords with the view that exosomes perform a role in cellular waste disposal (24). Regardless of the mechanism, our observation that these depolarization-associated vesicles were enriched with functionally significant primate-specific miRNA is fascinating and urges further investigation of exosomal release and transfer in the mammalian nervous system.

**Figure 7.**
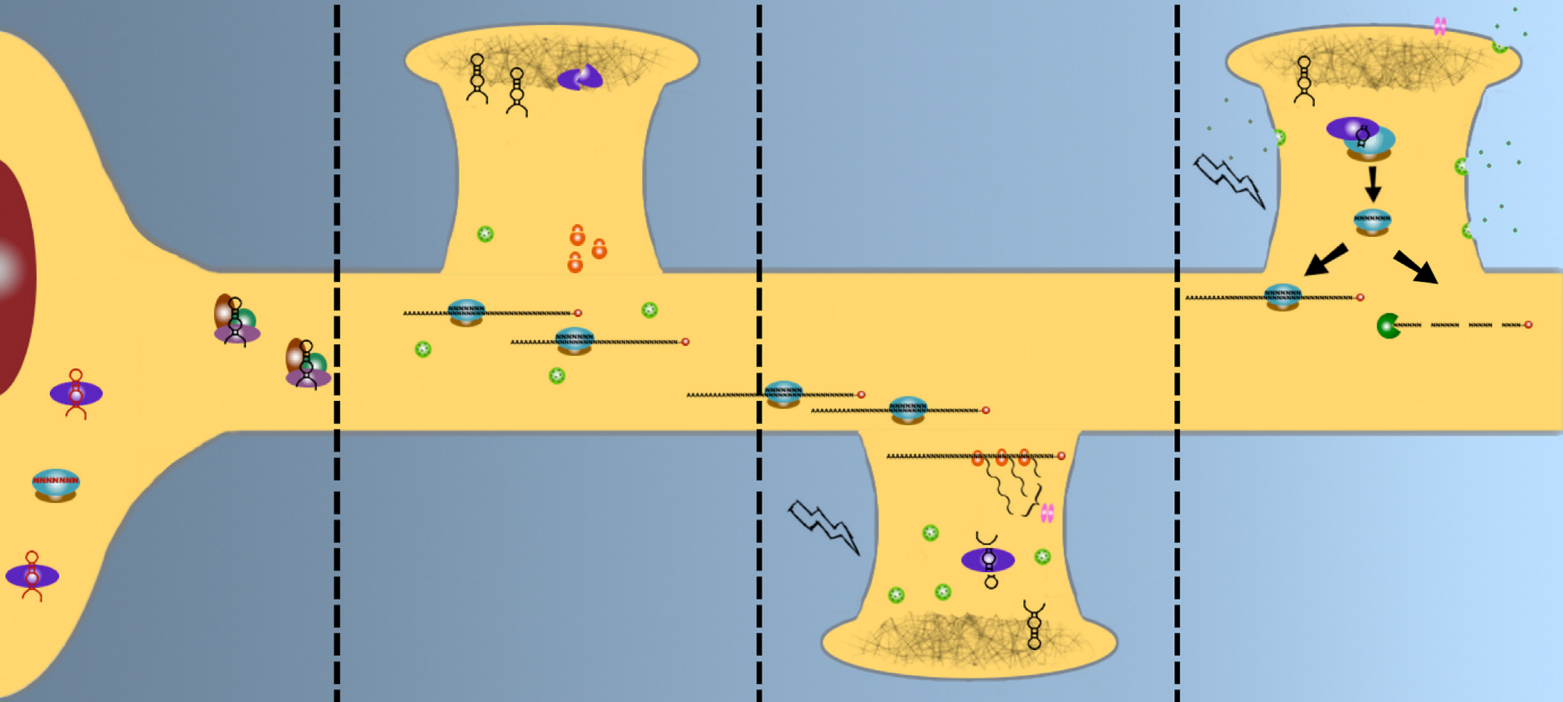
Integrated model of PTGS and synaptic plasticity. Dendritically-targeted miRNA (black) are sorted from cell body restricted transcripts (red), and transported either as precursor hairpins by RNP complexes (panel 1) for storage in the PSD along with inactive DICER (panel 2), or as part of an active RISC (panel 2). miRNA-enriched exosomes, packaged into multivesicular bodies (MVBs, green, panel 2), and ribosomes (orange) are scattered throughout the dendritic tree. When a spine is depolarized (panel 3), the RISC decompiles, freeing its mRNA cargo for translation, while DICER is activated and cleaves required hairpins from the PSD. As activity continues (panel 4), MVBs fuse with the cell membrane, releasing exosomes, while newly synthesized proteins are inserted into the membrane/PSD. The cleaved mature miRNA are loaded into an awaiting RISC, and may “mop-up” excess mRNAs to attenuate the response, either by silencing or degradation.

More broadly, the apparent functional specificity of miRNA compartmentalization, both in terms of their target analyses and the experimentally observed response to depolarization, lends support to the hypothesis that this process is involved with neural plasticity-associated translational regulation (Figure 7). In further support of this hypothesis, we recently demonstrated that while most target gene transcripts are inversely correlated with intracellular miRNA concentration, a substantial proportion are positively correlated (49). Functional annotation of the transcripts positively correlated with miRNA levels in SH-SY5Y cells suggested that these were involved in highly localized neural processes including neuroactive ligand-receptor interaction and adherens junctions. These observations further suggest that miRNA are delivered or generated with dynamic patterns of intracellular localization that enable them to collectively orchestrate even more complex combinatorial patterns of localized activity-dependent translation. Interestingly, the localization of the depolarization response to the neurite compartment was unique to miRNA, whereas mRNAs were modulated in both the neurites and cell bodies. Investigation of predicted targets of neurite-enriched, depolarization-responsive miRNA found enrichment of neuronally relevant genes, pathways and ontologies, suggesting that the expression changes identified are a deliberate response to stimulation of neuronally differentiating cells. Since both the up- and down-regulated cohorts are predicted to regulate expression of neuronal genes, these findings also suggest that depolarization selectively regulates the abundance of miRNA that directly modulate the response to this event and ultimately fine-tunes translational homeostasis.

In summary, we observed rapid depolarization-associated redistribution of miRNA in neurons, suggesting that they are an important regulatory component in the dynamics of normal synaptic function. As synaptic function is thought to be compromised in neurodegenerative and neuropsychiatric conditions, it is plausible that these molecules and their role in translational homeostasis are disrupted in these neuropathologies. This is supported by our postmortem investigation of schizophrenia, where we identified elevation of cortical miRNA expression that contrasted with the bias towards reduction of miRNA in response to depolarization observed in this study (22,50). If a reduction in PTGS is a requisite mechanism for synaptic plasticity and the over-abundance of miRNA in this pathology is insurmountable, the signal may be muted or lost. This mechanism warrants further investigation, as currently available therapeutics fail to provide improvement of cognitive symptoms. Interestingly, a large proportion of the depolarization-associated changes could be attributed to the release of exosomes containing synaptic protein MAP1b, and enriched with recently evolved miRNA. While the full implications are yet to be determined, it is tempting to speculate that these molecules and the associated mechanism could be involved in facilitating greater synaptic complexity and cognitive efficiency.

## SUPPLEMENTARY DATA

Supplementary Data are available at NAR Online.

## FUNDING

This study was supported by the Schizophrenia Research Institute utilising funding from NSW Health and an M.C. Ainsworth Research Fellowship in Epigenetics (MC); and Australian Postgraduate Award (BG); a NARSAD Young Investigator Award; and an NHMRC project grant APP1067137.

*Conflict of interest statement*. None declared.

## Supplementary Material

SUPPLEMENTARY DATA
