# Supplementary material for: Activity-associated miRNA are packaged in Map1b-enriched exosomes released from depolarized neurons
Source: Nucleic Acids Res. 2014 Jul 22;42(14):9195–208. doi: 10.1093/nar/gku594 (PMC4132720; doi:10.1093/nar/gku594)
Supplement: SUPPLEMENTARY DATA [file supp_42_14_9195__index.html]

Activity-associated miRNA are packaged in Map1b-enriched exosomes released from depolarized neurons — Activity-associated miRNA are packaged in Map1b-enriched exosomes released from depolarized neurons — SUPPLEMENTARY DATA 

# Activity-associated miRNA are packaged in Map1b-enriched exosomes released from depolarized neurons

## SUPPLEMENTARY DATA

**Files in this Data Supplement:**

- SUPPLEMENTARY DATA
