# Supplementary material for: Activity-associated miRNA are packaged in Map1b-enriched exosomes released from depolarized neurons
Source: Nucleic Acids Res. 2014 Jul 22;42(14):9195–208. doi: 10.1093/nar/gku594 (PMC4132720; doi:10.1093/nar/gku594)
Supplement: SUPPLEMENTARY DATA [file supp_gku594_nar-01320-y-2014-File009.zip › NAR-01320-2014 Suppl files/Goldie_et_al_Supplementary_Figure_Legends.docx]

**Figure S1. qPCR validation of neurite fractionation.** Known neurite markers synaptophysin (SYP) and growth-associated protein 43 (GAP43) were probed by qPCR of mRNA extracted from neurite (chequered bars) and cell body (open bars) fractions. Differential expression was calculated by the **ΔΔ**Ct method, using the cell body as control and comparing the neurite expression in the same biological sample. Data shown are from t-tests conducted on triplicate samples, graphing mean±sem. Both transcripts were significantly enriched in the neurites, confirming the success of the fractionation procedure.

**Figure S2. LAMP1 mRNA is enriched in neurites compared to cell bodies.** Relative abundance of LAMP1 mRNA in neurites and cell bodies of SH-SY5Y neuroblastoma was compared by qPCR using the **ΔΔ**Ct method as already described. (a) Neurites contained significantly more LAMP1 mRNA than cell bodies (p=0.0139, 1-tailed t-test). (b) LAMP1 mRNA was depleted by 1 and 4 successive K^+^ depolarisation/s.

**Figure S3. MAP1B-centred neuronal network connects exosome proteins.** Functional analysis of exosomal proteins was carried out in Integrated Pathways Analysis (IPA) software. A number of these proteins were found to have strong association with many aspects of neuronal biology, including number, shape and connectivity.

**Figure S4. Involvement of genes from the “G-protein coupled receptor signalling” pathway in the response to LTP-inducing stimulation.** (a) “G-protein coupled receptor signalling” and (b) “cAMP signalling” pathways were significantly over-represented by genes up- (red) and down- (green) regulated by LTP-inducing KCl stimulation.

**Figure S5. Late-phase LTP-associated “DNA Transcription” regulatory network.** miRNA down-regulated by 4-stimuli compared to 1 were target matched with mRNAs up-regulated at least 1.5-fold in the same condition. Functional analysis of this module showed a highly connected network involved in DNA transcription (red), which could be tightly regulated by miR-506 (yellow) and miR-548 (green). In white, miRs-22, -221 and -8 could also assist in regulating some aspects of this network.

**Figure S6. Neurite-specific “Neuronal Signalling” network.** Integrated Pathways Analysis (IPA) software was used to analyse functional modular connectivity between miRNA and mRNAs responding to depolarisation in the neurites of SH-SY5Y. A miRNA target analysis was integrated with experimental mRNA expression data; negatively correlated pairings were then filtered based on functionality. This analysis revealed a highly connected network comprising 4 miRNA (red) and many neurotransmitter receptors with negatively correlated expression changes (mRNA up, miRNA down) in response to a single stimulus.
