# Supplementary material for: Activity-associated miRNA are packaged in Map1b-enriched exosomes released from depolarized neurons
Source: Nucleic Acids Res. 2014 Jul 22;42(14):9195–208. doi: 10.1093/nar/gku594 (PMC4132720; doi:10.1093/nar/gku594)
Supplement: SUPPLEMENTARY DATA [file supp_gku594_nar-01320-y-2014-File009.zip › NAR-01320-2014 Suppl files/Goldie_et_al_Supplementary_Tables.docx]

**Table S1. Exosomal proteins depleted from cells by depolarisation.**

| Cells Only | Cells and Exosomes | Exosomes Only |
| --- | --- | --- |
| AKA12 | MAP1B | FLNA |
| KALRN | PRKDC | FLNB |
| TRRAP | DYHC1 | TPR |
|  | CLH1 |  |
|  | AHNAK |  |
|  | HTT |  |
|  | PLEC |  |
