# Supplementary figures and images for: Activity-associated miRNA are packaged in Map1b-enriched exosomes released from depolarized neurons
Source: Nucleic Acids Res. 2014 Jul 22;42(14):9195–208. doi: 10.1093/nar/gku594 (PMC4132720; doi:10.1093/nar/gku594)

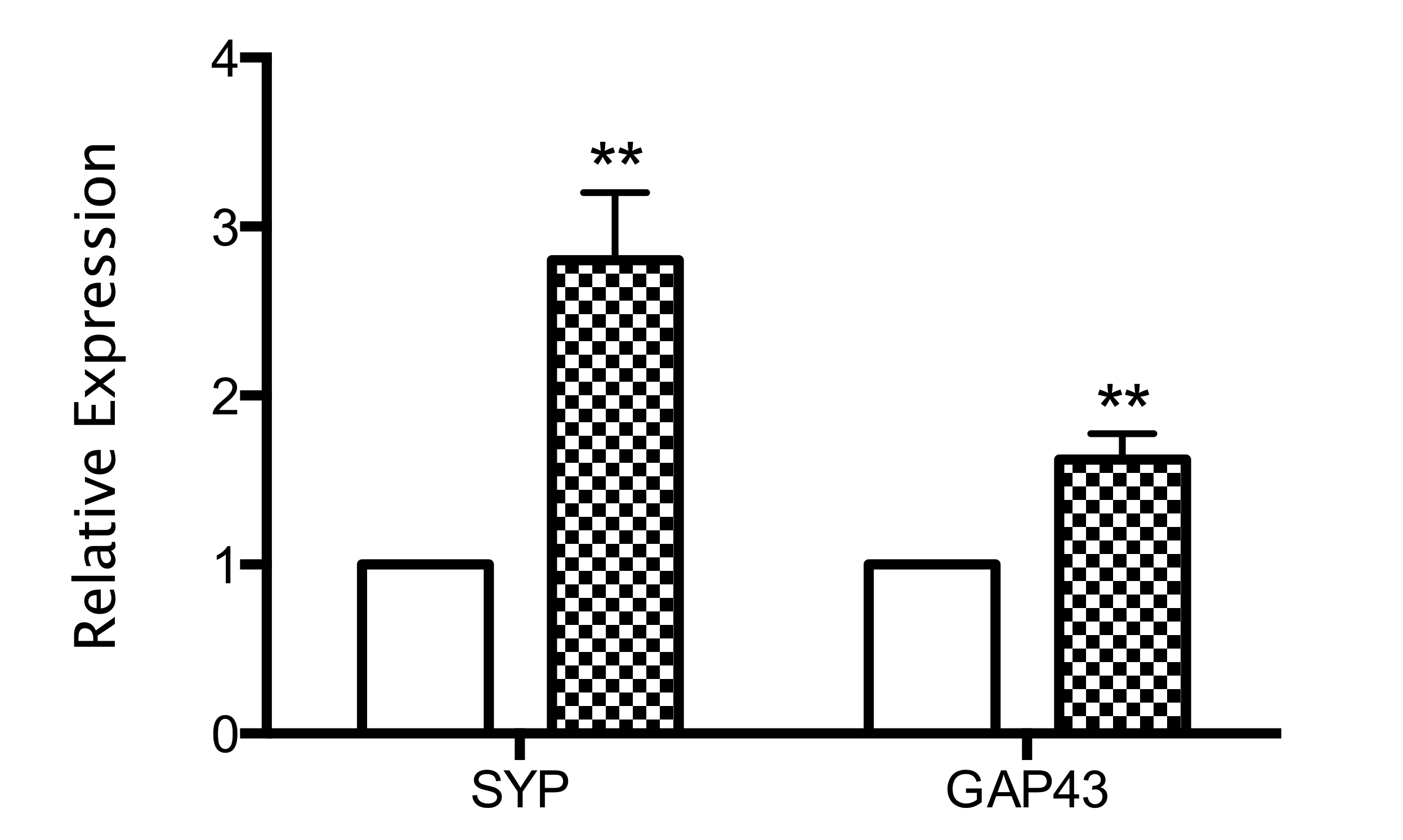

Supplement: SUPPLEMENTARY DATA [file supp_gku594_nar-01320-y-2014-File009.zip › NAR-01320-2014 Suppl files/Figure_S1.tiff]

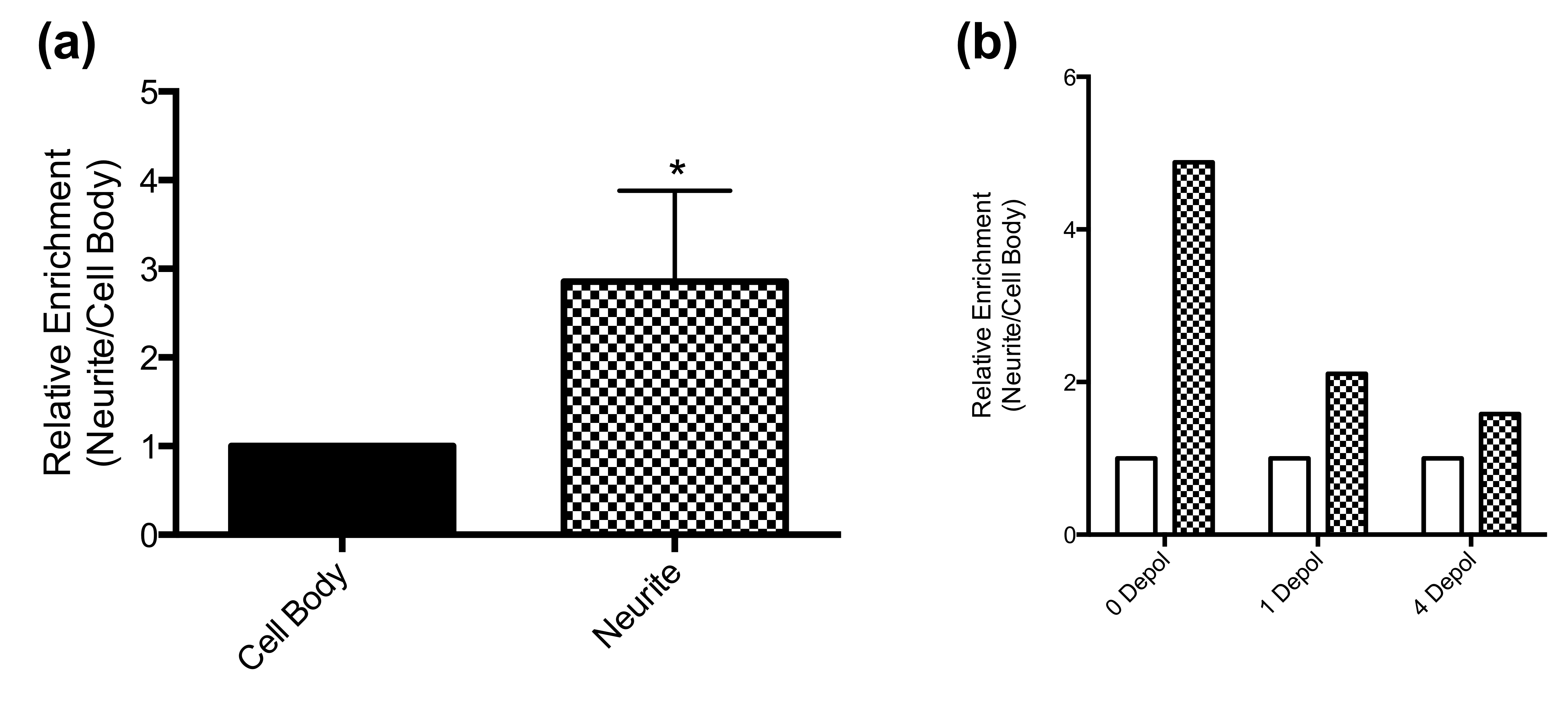

Supplement: SUPPLEMENTARY DATA [file supp_gku594_nar-01320-y-2014-File009.zip › NAR-01320-2014 Suppl files/Figure_S2.tiff]

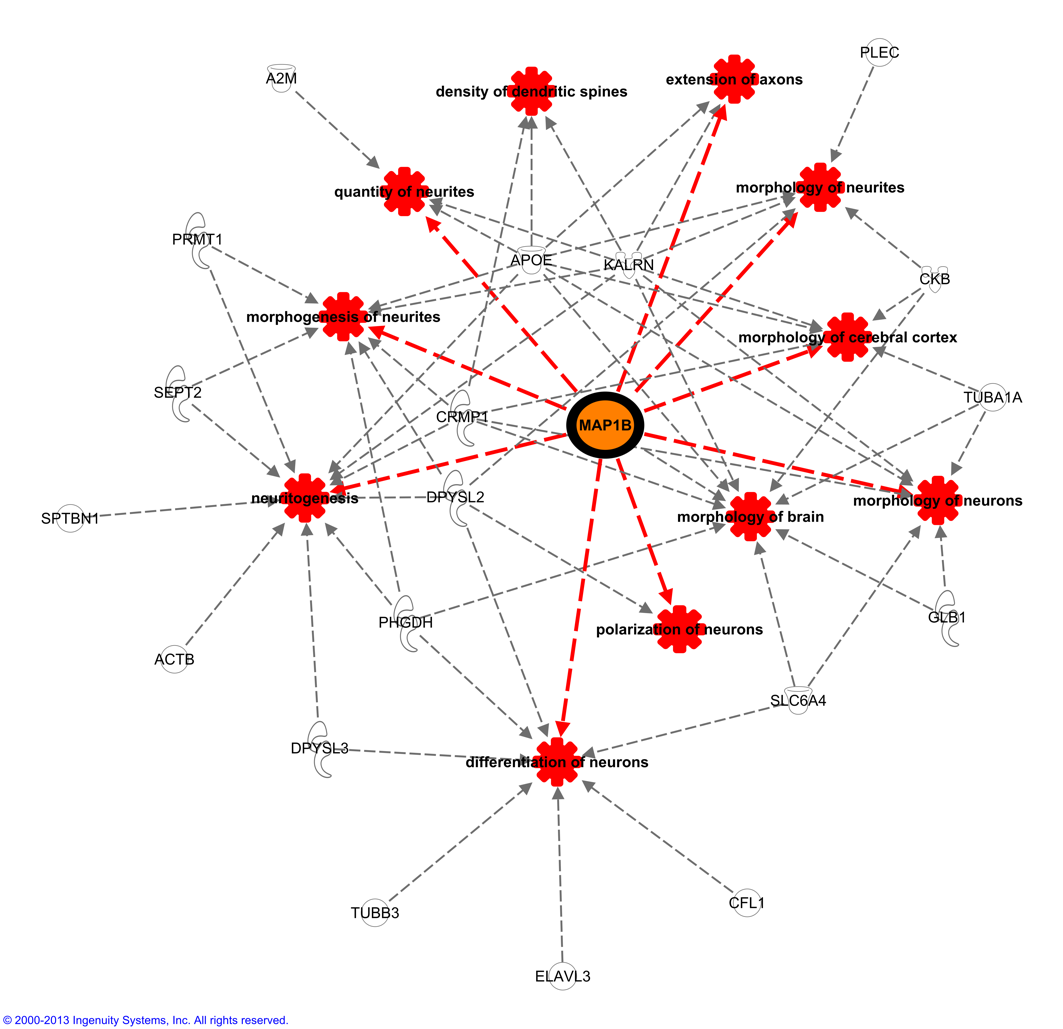

Supplement: SUPPLEMENTARY DATA [file supp_gku594_nar-01320-y-2014-File009.zip › NAR-01320-2014 Suppl files/Figure_S3.tiff]

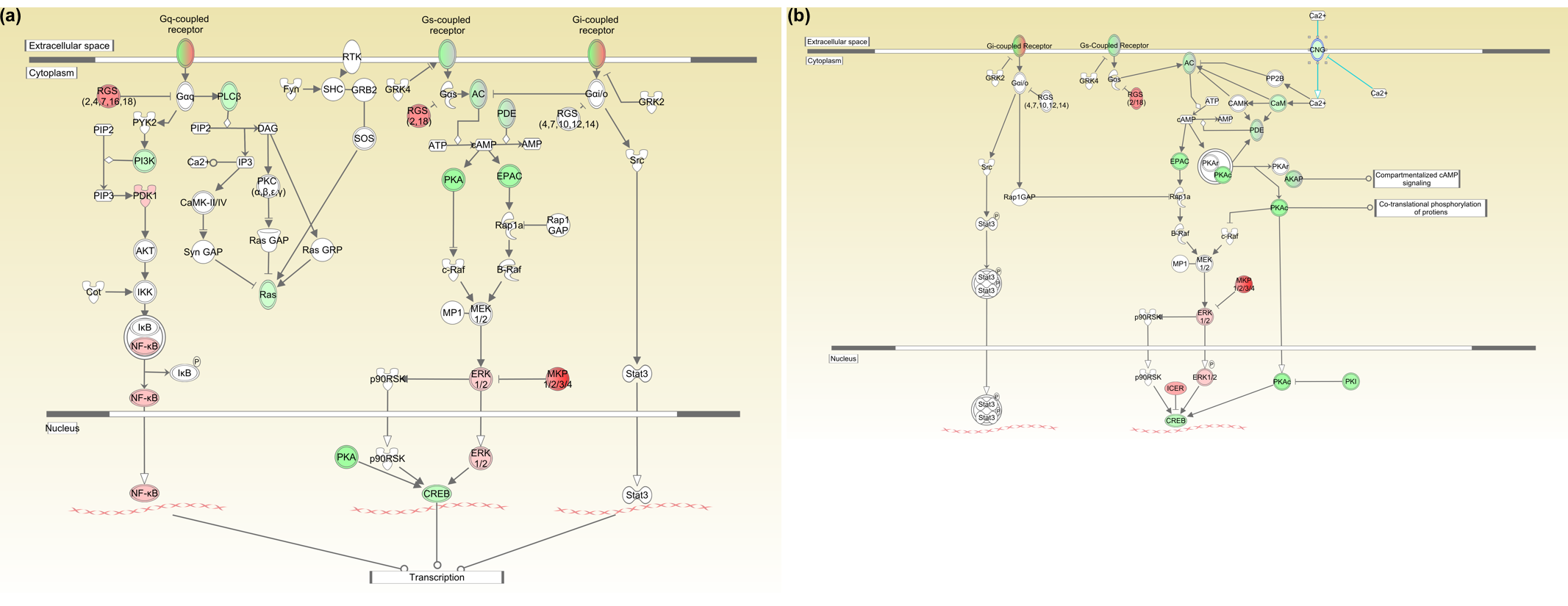

Supplement: SUPPLEMENTARY DATA [file supp_gku594_nar-01320-y-2014-File009.zip › NAR-01320-2014 Suppl files/Figure_S4.tiff]

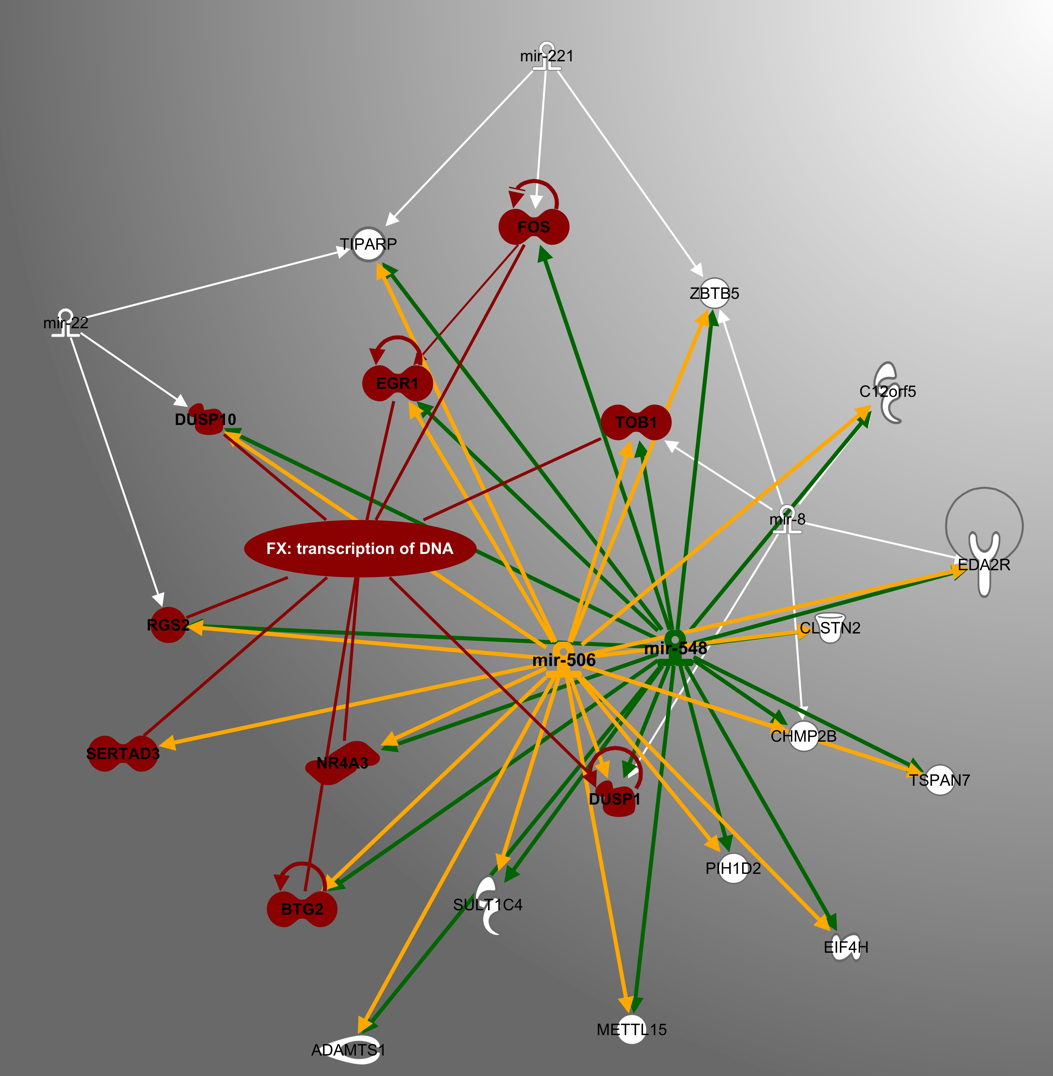

Supplement: SUPPLEMENTARY DATA [file supp_gku594_nar-01320-y-2014-File009.zip › NAR-01320-2014 Suppl files/Figure_S5.tiff]

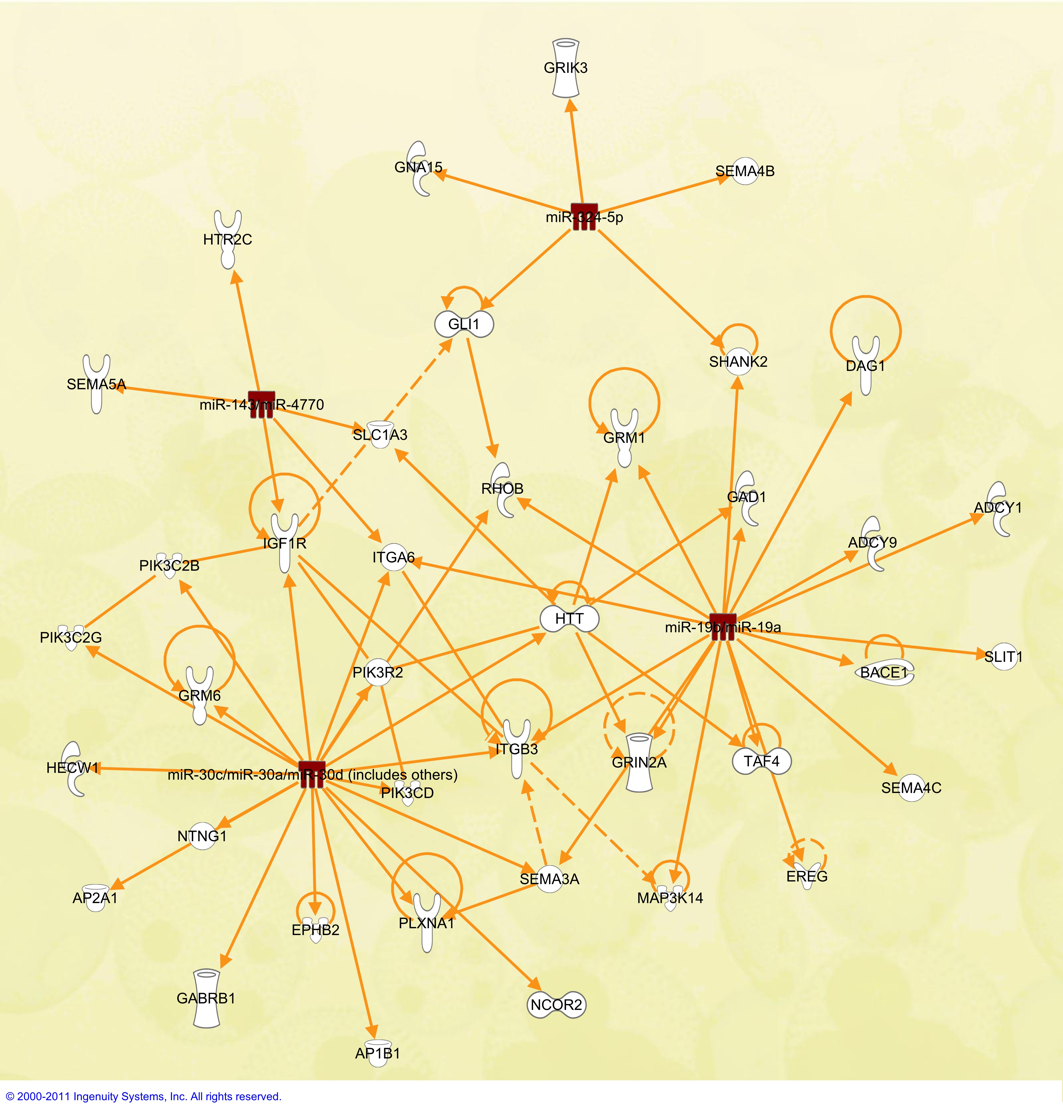

Supplement: SUPPLEMENTARY DATA [file supp_gku594_nar-01320-y-2014-File009.zip › NAR-01320-2014 Suppl files/Figure_S6.tiff]
